# The Neuroprotective Effects of Carvacrol on Ethanol-Induced Hippocampal Neurons Impairment via the Antioxidative and Antiapoptotic Pathways

**DOI:** 10.1155/2017/4079425

**Published:** 2017-01-16

**Authors:** Peng Wang, Qian Luo, Hui Qiao, Hui Ding, Yonggang Cao, Juan Yu, Ruxia Liu, Qianlong Zhang, Hui Zhu, Lihui Qu

**Affiliations:** ^1^Department of Physiology, College of Basic Medical Sciences, Harbin Medical University, Harbin 150081, China; ^2^Department of Physiology, College of Basic Medical Sciences, Harbin Medical University-Daqing, Daqing 163319, China; ^3^Department of Gastroenterology, Daqing Oilfield General Hospital, Daqing 163319, China; ^4^Department of Anesthesiology, Daqing Fifth Hospital, Daqing 163319, China; ^5^Department of Pharmacology, College of Basic Medical Sciences, Harbin Medical University-Daqing, Daqing 163319, China

## Abstract

Chronic alcohol consumption causes hippocampal neuronal impairment, which is associated with oxidative stress and apoptosis. Carvacrol is a major monoterpenic phenol found in essential oils from the family Labiatae and has antioxidative stress and antiapoptosis actions. However, the protective effects of carvacrol in ethanol-induced hippocampal neuronal impairment have not been fully understood. We explored the neuroprotective effects of carvacrol in vivo and in vitro. Male C57BL/6 mice were exposed to 35% ethanol for 4 weeks to establish ethanol model in vivo, and hippocampal neuron injury was simulated by 200 mM ethanol in vitro. Morris water maze test was performed to evaluate the cognitive dysfunction. The oxidative stress injury of hippocampal neurons was evaluated by measuring the levels of oxidative stress biomarkers. Histopathological examinations and western blot were performed to evaluate the apoptosis of neurons. The results showed that carvacrol attenuates the cognitive dysfunction, oxidative stress, and apoptosis of the mice treated with ethanol and decreases hippocampal neurons apoptosis induced by ethanol in vitro. In addition, western blot analysis revealed that carvacrol modulates the protein expression of Bcl-2, Bax, caspase-3, and p-ERK, without influence of p-JNK and p-p38. Our results suggest that carvacrol alleviates ethanol-mediated hippocampal neuronal impairment by antioxidative and antiapoptotic effects.

## 1. Introduction

It is well known that ethanol is a deleterious agent which can damage many organs and cause serious health problems [1–4]. Long-term excessive consumption of ethanol leads to behavioral changes, addiction, hyperactivity, mental retardation, depression, and cognitive dysfunction [5–7]. Studies demonstrated that ethanol exposure reduces hippocampal volume, decreases glucose metabolism of cerebrum, and cerebral blood flow and has effects on several neurotransmitter systems that may contribute to cognitive deficits [8–12]. However, less is known about the detailed mechanism of the effects of ethanol on hippocampal neurons damage. Oxidative stress has been considered as the most plausible cause of ethanol-induced neuronal damage [13–15]. Ethanol promotes production of lipid peroxidation, increases reactive oxygen species (ROS), decreases the activity of antioxidant enzymes, and augments oxidative stress [16–18]. Furthermore, the imbalance of oxidation and antioxidation activates apoptotic cascades by mitochondrial signaling pathway [19–21]. In addition, cumulative evidences indicated that ethanol-induced oxidative stress also participates in the modulation of the mitogen-activated protein kinase (MAPK) pathways [22, 23].

Carvacrol [CAR, C_6_H_3_(OH)(C_3_H_7_)] is a natural component found in various plants of the family Lamiaceae, including the genera* Origanum* and* Thymus*. [24]. It has been widely used as an ingredient of human diet in food or food additive in the food industry for long time. In recent years, several studies have reported that carvacrol has multiple physiological actions, including antibacterial and antifungal [25, 26], anti-inflammatory [27], and antitumor activities [28].

Aside from these above pharmacological effects, potential protective effect of carvacrol also has been observed to the central nervous system diseases through diverse mechanisms, including anxiolytic effect, and inhibitory effect on acetylcholinesterase activity [29]. In addition, a recent study demonstrated that carvacrol presented its neuroprotective effect against cerebral ischemia/reperfusion injury in mice [30]. However, there are no detailed reports regarding a potential protective effect of carvacrol against ethanol-mediated hippocampal neuronal impairment in C57BL/6 mice. Therefore, the present study was conducted to evaluate the neuroprotective potential of carvacrol against ethanol-mediated hippocampal neuronal impairment, as well as further explore whether neuroprotection of carvacrol was associated with antioxidation and antiapoptosis with the mitochondrial and MAPK signaling pathway involved.

## 2. Methods

### 2.1. Animals and Treatments

**Figure 1:**
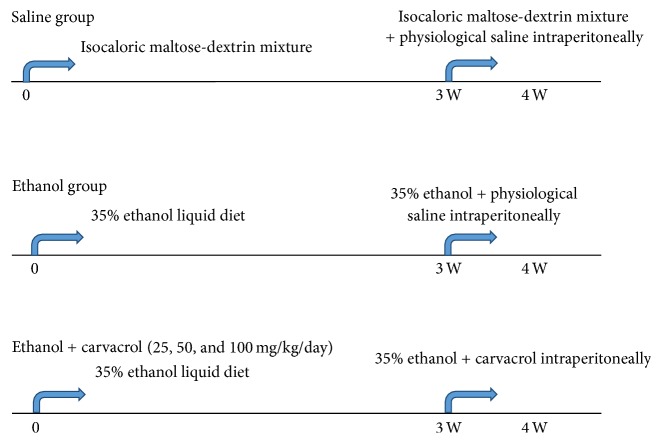
Scheme of the in vivo experimental design.

Male C57BL/6 mice (25 ± 0.5 g) were allowed to acclimatize (12 : 12 h light/dark cycle, 24°C) for 7 days. The use of the animal tissue was approved by the Ethics Committee of Laboratory Animals at Harbin Medical University. A summary of the experimental design is provided in Figure 1. Mice were randomly assigned to 2 groups including the control diet (saline group) and ethanol diet group. The groups were pair-fed liquid diets containing (in percent of energy intake) 18% protein, 35% fat, 12% carbohydrate, and 35% either ethanol (ethanol diet) in ethanol group or an isocaloric maltose-dextrin mixture (control diet) in saline group for 4 weeks [31]. Then, after 3 weeks of control diet treatment, saline group mice were treated with control diet and vehicle intraperitoneally (physiological saline 0.1 mL/100 g/day) for 7 days. The ethanol diet mice were divided into 4 groups as follows. (1) Ethanol group: mice were treated with ethanol diet for 3 weeks; the following 7 days mice were treated with ethanol diet and vehicle intraperitoneally (physiological saline 0.1 mL/100 g/day); (2) Ethanol + Carvacrol groups: mice were treated with ethanol diet for 3 weeks, and then the mice were subjected to ethanol diet and carvacrol intraperitoneally at 25, 50, and 100 mg/kg/day, respectively, for 7 days. After the endpoint of treatments, mice were used for the Morris water maze, morphological experiments, oxidative stress measurements, and western blot. Carvacrol (cat# W224502) was purchased from Sigma-Aldrich, USA.

### 2.2. Blood Ethanol Concentration

To determine blood ethanol concentration (BEC), blood was collected from tail once a week. Tail blood samples were centrifuged to obtain plasma, and the ethanol concentration was measured with an Analox AM1 analyzer (Analox Instruments; Lunenburg, MA, USA, cat# GMRD-023).

### 2.3. Morris Water Maze

After 4 weeks of ethanol consumption, 50 mice (*n* = 10) were used for Morris water maze (MWM) test. The experiment was performed in a white circular water tank (150 cm diameter and 60 cm height) with a smooth inner surface. It rendered opaque water at 22 ± 1°C with white synthetic food colors. A 10 cm square platform was located 2 cm below the water surface. The pool was divided into four quadrants, and the platform was placed at the midpoint of a quadrant. On the 1st day, all mice were allowed to swim freely. On the 2nd–4th day, the mice were pretrained to find the hidden platform. On the 5th–8th day, each mouse was subjected to 4 trails per day in a maximum of 60 s. The time to climb onto the platform was recorded for each trial as escape latency (s). On the 9th day, the platform was removed and the passing times of the mice that crossed the place where the platform was previously located were recorded.

### 2.4. Nissl Staining

After animals received ethanol or control diet 4 weeks, respectively, the mice in each group (*n* = 6) were anesthetized with 10% (v/v) chloral hydrate and transcardially perfused with 0.1 M phosphate buffered saline (PBS, pH 7.4) for 10 min, followed by fixation by 4% paraformaldehyde in 0.1 M phosphate buffered saline (PB, pH 7.4) for 10 min. The brains were then removed, postfixed in 4% paraformaldehyde for 48 h, and then cryoprotected by infiltration with 30% sucrose for 3 days at 4°C. Coronal sections (8 *μ*m) of the hippocampus were cut and submerged in 0.1% cresyl violet for 10 min at 37°C and then were rinsed in distilled water, dehydrated in graded ethanol, and cover lipped with neutral balsam. For Nissl staining, the normal neurons were round, and the nuclei appeared as pallid blue. In the ethanol-treated group, the cells were shrunken, with nuclei pyknosis. Quantitative assessment of the number of live neurons was evaluated from 10 CA1 region per brain sample (*n* = 6) by Image-Pro Plus 6.0 (Media Cybernetics, Bethesda, MA, USA).

### 2.5. NeuN Immunohistochemistry

Hippocampus injury was evaluated based on the results of Nissl staining and immunohistochemistry in brain sections. Tissue sections were treated with 0.3% hydrogen peroxide (H_2_O_2_) for 10 min and then nonspecific antibody binding was blocked with 10% goat serum for 30 min at room temperature. The sections were incubated with anti-NeuN (1 : 200, Chemicon, CA) overnight at 4°C, and, subsequently, the sections were exposed to biotinylated goat anti-mouse IgG and streptavidin peroxidase complex (Vector, Burlingame, CA) for 30 min at 37°C. They were soaked in 3,3-diaminobenzidine (DAB), and the reaction was stopped with distilled water. The stained sections were observed under a light microscope. Quantification of the number of NeuN-immunopositive cells from 10 CA1 region per brain sample (*n* = 6) was by Image-Pro Plus 6.0 (Media Cybernetics, Bethesda, MA, USA).

### 2.6. Double Immunofluorescence Staining

Immunofluorescent double staining of NeuN and TUNEL was performed to explore colocalization of apoptotic cells and neurons. The hippocampal tissue sections were treated the same way as experimental procedure in Section 2.4 before TUNEL and NeuN staining. TUNEL staining was conducted according to the manufacturer's protocol of In Situ Cell Death Detection Kit (Roche, Germany), followed by antibody staining against NeuN (1 : 200, Chemicon, CA). Finally, the sections were observed under a fluorescence microscope (Leica, Germany).

### 2.7. Hippocampal Neurons Culture

Neonatal rat hippocampal neurons were prepared from 2- to 3-day-old neonatal C57BL/6 mice. The hippocampi were dissected and chopped into 1 mm^3^ pieces under a light microscope. The hippocampal chunks were digested by 0.125% (w/v) trypsinase at 37°C for 10 min and then resuspended in DMEM supplemented 10% fetal bovine serum (FBS) in order to stop trypsin activity. After centrifugation at 14,000*g* for 5 min, the supernatant was discarded and the cell pellet was resuspended in DMEM. Hippocampal cultures were maintained in serum-free Neurobasal medium, containing B27 supplement in a humidified atmosphere (95% air, 5% CO_2_) at 37°C. Hippocampal neurons were chosen for experiments between days 8 and 14 of culture. Hippocampal neurons were randomly divided into 4 groups: (1) control group; (2) model group, cells were incubated with 200 mM ethanol for 24 h; (3) model + carvacrol group, cells were given 0.8 mM carvacrol for 4 h after treatment with 200 mM ethanol for 24 h; (4) control + carvacrol group, cells were given 0.8 mM carvacrol for 4 h.

### 2.8. Hippocampal Neurons Viability Assay

Hippocampal neurons were plated at 30,000 cells/well in a 96-well plate. Cell viability was assessed at day 2 of cell culture. According to the manufacturer's recommendations, 40 *μ*L MTS solution was added to each of the wells, and absorbance was obtained at 490 nm using a microplate reader (FlexStation 3; Molecular Devices, Sunnyvale, CA, USA). The same volume of medium without cells was used as blank.

### 2.9. Hoechst Staining

Hippocampal neurons were grown in six-well plates. After drug treatments, cells were fixed with 4% paraformaldehyde for 4°C overnight. Fixed cells were washed with PBS three times and stained with Hoechst 33258 (final concentration, 0.5 ug/mL) for 5 min. The six-well plates were visualized using a fluorescent microscope (IX51, Olympus).

### 2.10. Oxidative Stress Measurements

Mice were sacrificed after treatment of ethanol or control diet for 4 weeks, respectively. The hippocampus was rapidly excised from the brain and kept frozen at −80°C. Half of the tissue was taken for oxidative stress measurements and the other half of the section was detected for western blot analysis. The enzymatic activities of CuZn-superoxide dismutase (SOD), Mn-SOD, SOD, glutathione (GSH), glutathione peroxidase (GSH-PX), and catalase (CAT) and the level of malondialdehyde (MDA) were evaluated with different detection kits according to the manufacturer's instructions (Nanjing Jian Cheng Bioengineering Institute). One part of the tissues which was used for oxidative stress measurements was weighed and rinsed with cold isotonic saline. Hippocampus homogenate (10%, w/v) was prepared by homogenizing the hippocampus tissue in cold saline (pH 7.0). CuZn-SOD and Mn-SOD activities were detected in hippocampus homogenate by measuring the ability to inhibit the photochemical reduction of nitro blue tetrazolium (NBT) in absorbance at 550 nm. Data were recorded as SOD units/mg protein. The level of MDA in the hippocampus was calculated by measuring thiobarbituric acid reacting substances at 532 nm. The concentration of malondialdehyde was expressed as nmol/mg protein. The activities of GSH-PX and GSH were detected by quantifying the rate of reduced glutathione to the oxidized glutathione. The levels of enzymatic activities were measured with a spectrophotometer (U-2000, Hitachi).

### 2.11. Western Blot

Western blot analysis was performed on the hippocampal tissues. Briefly, tissues were lysed with RIPA buffer (Beyotime Institute of Biotechnology, China) containing 50 mM Tris, 150 mM NaCl, 1% Triton X-100, 1 mM EDTA, and 2 mM PMSF containing protease inhibitors for 30 min on ice. The lysates were centrifuged at 13,500*g* for 15 min at 4°C, and the supernatant was collected and total protein content was determined using a BCA protein assay kit with bovine serum albumin as the standard (Beyotime Institute of Biotechnology, China). Samples with an equal amount of protein (50 *μ*g) were separated in 10% or 12% SDS-polyacrylamide gels and then transferred onto nitrocellulose membranes (Millipore, MA). The membranes were then blocked using 5% fat-free milk in Tris-buffered saline with 1% Tween-20 (TBS-T) for 1 h and then were incubated with the following incubation with primary antibodies overnight at 4°C: anti-caspase-3 (1 : 300, Santa Cruz), anti-Bcl-2 (1 : 200, Santa Cruz), anti-Bax (1 : 200, Santa Cruz), anti-phospho-ERK (1 : 1000, Cell Signaling), anti-ERK (1 : 1000, Cell Signaling), anti-phospho-JNK (1 : 200, Santa Cruz), anti-JNK (1 : 200, Santa Cruz), anti-phospho-p38 (1 : 200, Santa Cruz), anti-p38 (1 : 200, Santa Cruz), and anti-*β*-actin (1 : 2000, Santa Cruz), respectively. The membranes were washed with TBS-T, followed by the incubation with horseradish peroxidase-conjugated goat anti-rabbit-antibody (1 : 5000, Santa Cruz) or goat anti-mouse antibody (1 : 5000, Santa Cruz), for 2 h at room temperature. Immunoreactive bands were visualized by enhanced chemiluminescence (ECL) kit (Pierce, CA) and exposed on an X-ray film. The immunoblots intensities were quantified using the Quantity One software (BioRad).

### 2.12. siRNA Transfection

To silence the expression of ERK1/2 protein, hippocampal neurons were transfected with small interfering RNA, which was designed and synthesized by GenePharma (Shanghai, China). Nontargeted control siRNA (si-NC) was used as negative control. The sense sequence of siRNA against ERK1/2 and nontargeted control sequence were listed below: accession No.ds-siRNA sequence corresponding nucleotides, ERK1: 5′-G G A C C A G C U C A A C C AC A U U-3′, ERK2: 5′-G C U C U U G A A G A C A C A G C A C-3′ NC control: 5′-UUCUCCGAACGUGUCACGUTT-3′. Briefly, the hippocampal neurons were cultured till 30%–50% confluence and then 2 ug siRNA and 10 ul X-tremeGene siRNA Transfection Reagent were, respectively, diluted in serum-free Opti-MEM-1 medium and mixed. The mixture (siRNA/Transfection Reagent) was incubated at room temperature for 20 min and added directly onto cells. After transfections, cells were quiesced for 48 h and used as required.

### 2.13. Flow Cytometric Analysis

The apoptosis rate of hippocampal neurons was carried out by flow cytometric analysis. Both attached and floating hippocampal neurons were harvested and washed twice with PBS, fixed in ice-cold 80% ethanol, and stored overnight at 4°C. Then, the cells were washed twice with PBS and 10 mg/mL RNase A was added. FITC-labeled Annexin V/PI staining was performed according to the manufacturer's instructions (Keygen, Nanjing, China). For each experiment, 20,000 cells were analyzed using Elite Flow Cytometry (BD Biosciences, San Jose, CA) and Cell Quest software (BD Biosciences). Triplicates were performed in all cases for the detection of early apoptotic cells.

### 2.14. Caspase-3 Activity Assay

A colorimetric assay kit (Beyotime, Jiangsu, China) was used to evaluate the activity of caspase-3. Briefly, hippocampal neurons were treated the same way as experimental procedure in Section 2.11 and then were centrifuged at 4°C for 20 min, the supernatant was collected. Approximately 1–3 *μ*g/mL protein was incubated with substrate Ac-DEVD-pNA (acetyl-Asp-Glu-Val-Aspnilide) at 37°C for 2 h. The absorbance of pNA was measured with a spectrophotometer at 405 nm. The activity of caspase-3 was normalized for total protein and expressed as fold of the baseline caspase-3 activity of the control group.

### 2.15. Statistical Analysis

Data were represented as mean ± SEM. Statistical analysis was carried out using one-way ANOVA followed by Turkey test for individual comparisons between group means. All statistical analyses were performed by SPSS 19.0 software. *P* < 0.05 was considered statistically significant.

## 3. Results

### 3.1. Effect of Carvacrol on Body Weight and Blood Ethanol Levels

**Figure 2:**
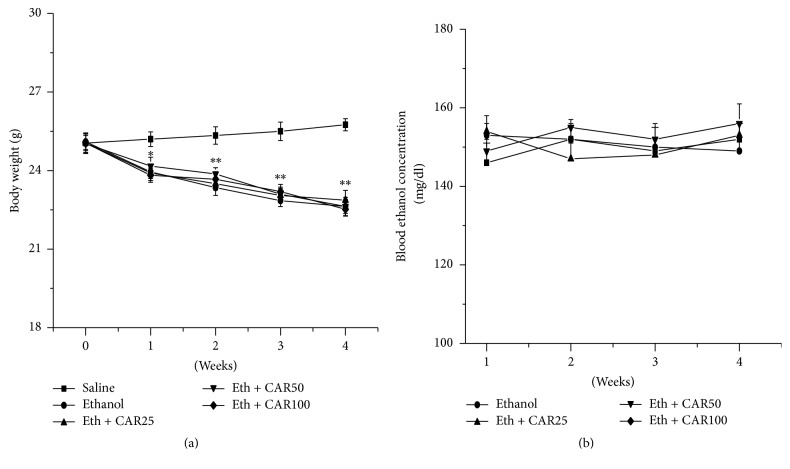
(a) Body weight changes of mice during 4 weeks liquid diets treatment. No significant differences were detected between ethanol and ethanol + CAR (25, 50, and 100 mg/kg/day) groups. (b) Blood ethanol concentration in different groups of mice during 4 weeks treatment. No significant effect of carvacrol and no significant interaction between ethanol and carvacrol in blood ethanol concentration. The data are expressed as the mean ± SEM (*n* = 6 per group). ^*∗*^*P* < 0.05 and ^*∗∗*^*P* < 0.01 compared to saline group.

4 weeks after ethanol diet, mice exhibited significantly decreased body weights compared with the saline group (*P* < 0.05). However, chronic treatments with carvacrol at the doses of 25, 50, and 100 mg/kg displayed no influence with the blood ethanol levels and body weights of mice, compared with the ethanol group (*P* > 0.05) (Figures 2(a) and 2(b)). The results indicated that carvacrol does not affect blood ethanol concentration and body weight of mice.

### 3.2. Carvacrol Improved Ethanol-Induced Cognitive Deficits

**Figure 3:**
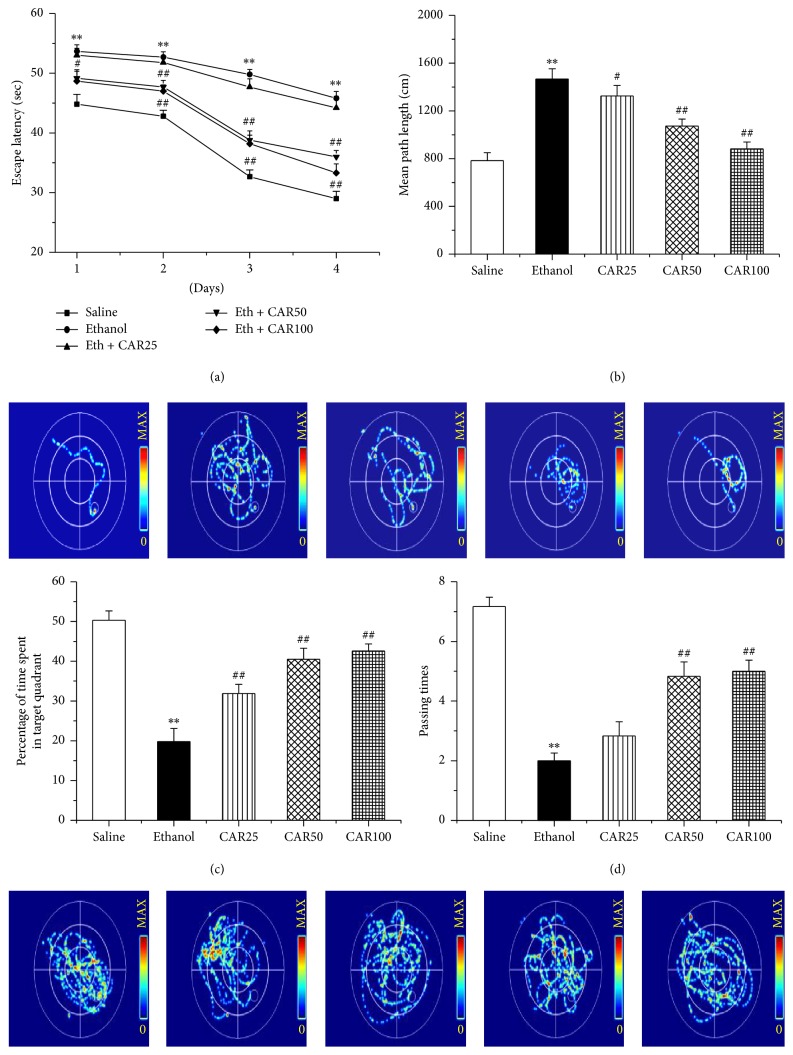
Carvacrol ameliorated ethanol-induced cognitive impairment in C57BL/6 mice. Escape latency (a), mean path length (b), mice that stayed in the target quadrant (c), and passing times (d) are shown in the saline, ethanol, and different carvacrol dose groups. CAR25, CAR50, and CAR100 indicate carvacrol 25, 50, and 100 mg/kg-treated groups. The data are expressed as the mean ± SEM (*n* = 10 per group). ^*∗∗*^*P* < 0.01 compared to saline group; ^#^*P* < 0.05 and ^##^*P* < 0.01 compared to ethanol group.

Cognitive function was assessed with the Morris water maze test. By one-way ANOVA analysis, as shown in Figure 3(a), the escape latency of the ethanol-treated group significantly increased in comparison to that in the saline group (*P* < 0.01), while carvacrol treated animals at dose of 50 and 100 mg/kg reversed this effect (*P* < 0.01). There was no significant difference between ethanol and carvacrol 25 mg/kg treatment group (*P* > 0.05). Likewise, Figure 3(b) also indicated an evident increase in mean path length in ethanol-treated group; this phenomenon was obviously prevented after treatment with carvacrol in a dose-dependent way (*P* < 0.01). The evident decreases for the time stayed in the target quadrant and the number of times the animals crossed the former platform location were observed in ethanol-treated group versus saline group (*P* < 0.01), as shown in Figures 3(c) and 3(d), respectively. However, supplement with carvacrol (50 and 100 mg/kg) dose-dependently reversed the two indices (*P* < 0.01).

### 3.3. Carvacrol Protected Hippocampal Neuron against Ethanol-Induced Apoptosis

**Figure 4:**
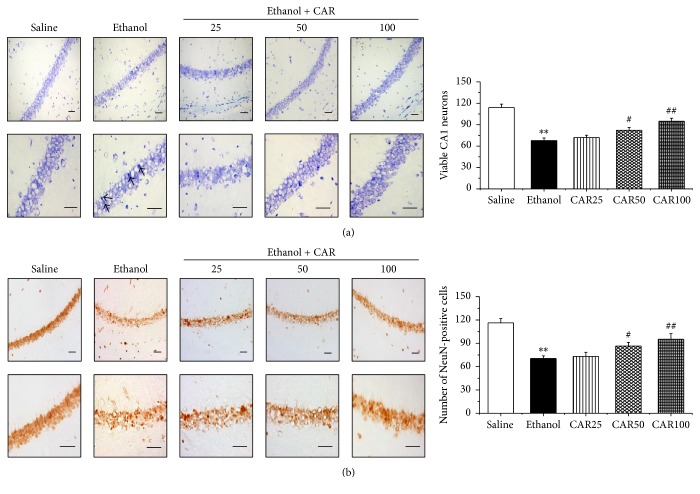
Nissl staining and NeuN immunohistochemistry showed the protective effects of carvacrol on ethanol-induced neuronal impairment of the hippocampal CA1 region in C57BL/6 mice. (a) Representative photomicrographs of Nissl staining for surviving neurons in hippocampal CA1 region, and the statistical analysis of the surviving cells in each group. Arrows indicated the shrunk dark damaged neurons. (b) Representative immunohistochemical photomicrographs of NeuN in mouse hippocampus, and the statistical analysis of the NeuN-immunopositive cells in each group. There were fewer NeuN and Nissl-positive neurons in the ethanol group than in the saline group. With treatment with different doses of carvacrol, NeuN and Nissl-positive neurons were abundant in the CA1 region compared with the ethanol group. Up panel is lower magnification image (200x) and down panel is higher magnification image (400x) of CA1 pyramidal neurons. Scale bar: 50 *μ*m. The data are expressed as the mean ± SEM (*n* = 6 per group). ^*∗∗*^*P* < 0.01 compared to saline group; ^#^*P* < 0.05 and ^##^*P* < 0.01 compared to ethanol group.

**Figure 5:**
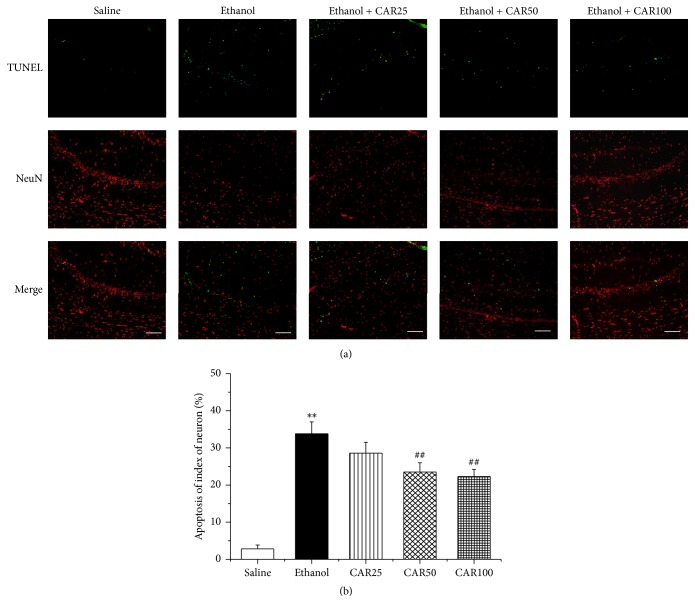
Double fluorescent labeling TUNEL (green) and NeuN (red) showed the protective effects of carvacrol on ethanol-induced neuronal impairment of the hippocampal CA1 region in C57BL/6 mice. (a) Representative photographs of TUNEL and NeuN staining in different groups. TUNEL-positive cells were barely detected in the saline group. In the ethanol group, the apoptotic cells increased markedly compared with the saline group. However, compared with the ethanol group, administration of different doses of carvacrol substantially reduced the number of apoptotic cells. (b) Quantification of TUNEL-positive cells. Scale bar: 100 *μ*m. The data are expressed as the mean ± SEM (*n* = 6 per group). ^*∗∗*^*P* < 0.01 compared to saline group; ^##^*P* < 0.01 compared to ethanol group.

In order to explore the effects of carvacrol on neuron apoptosis induced by chronic ethanol feeding in hippocampal tissues, histological examination of Nissl staining was performed to evaluate a significant loss of ethanol-induced hippocampal CA1 region neurons. As illustrated in Figure 4(a), neurons in the saline group did not have any histopathological abnormalities; however, extensively damaged neurons with pyknotic nuclei were observed in ethanol-treated mice. Carvacrol treatment markedly increased cell survival with palely stained nuclei in comparison with the ethanol group (*P* < 0.01). NeuN immunohistochemistry showed that the number of NeuN-immunopositive cells in the hippocampal CA1 region was evidently decreased in the ethanol group compared with the saline group as shown in Figure 4(b). Carvacrol treatment animals at doses of 50 and 100 mg/kg markedly prevented ethanol-induced neuronal damage (*P* < 0.01). Double-label staining of TUNEL and NeuN showed that the number of NeuN-TUNEL-positive cells was increased in the ethanol group compared with the saline group (*P* < 0.01). The ethanol group mice treated with carvacrol had significantly (*P* < 0.01) less NeuN-TUNEL-positive cells in the hippocampal CA1 region than the mice in ethanol group had (Figure 5).

**Figure 6:**
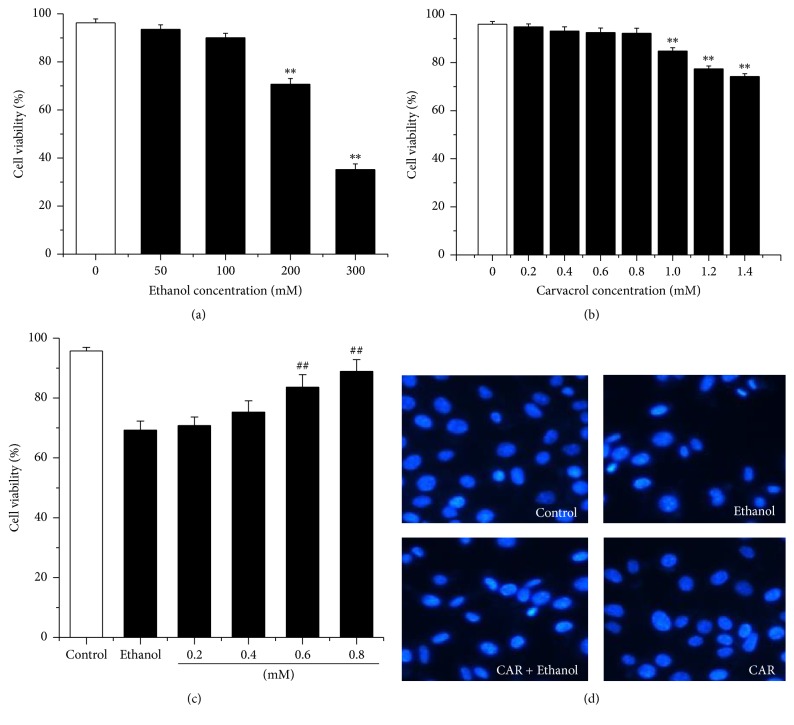
Carvacrol protects cultured hippocampal neurons from ethanol-induced cell death. (a)–(c) To select drug concentrations for the subsequent experiments, MTT assay was carried out on the hippocampal neurons. (a) Primary hippocampal neurons were treated with 0–300 mM ethanol. (b) Hippocampal neurons were treated with 0–1.4 mM carvacrol. (c) Hippocampal neurons were treated with 200 mM ethanol and then exposed to different doses of carvacrol. On the basis of the above results, hippocampal neurons treated with 0.8 mM carvacrol after the incubation of 200 mM ethanol were selected. (d) Hippocampal neurons were incubated with 0.8 mM carvacrol after the incubation of 200 mM ethanol. Cells were observed by fluorescence microscopy after the nuclei were stained with the fluorescent dye Hoechst 33342. All the values are presented as mean ± SEM (*n* = 6 per group). ^*∗∗*^*P* < 0.01 compared to control group; ^##^*P* < 0.01 compared to ethanol group.

To detect the antiapoptosis of carvacrol against ethanol toxicity in hippocampal neurons, the best concentrations of carvacrol and ethanol were explored using MTT assay. As shown in Figure 6(a), hippocampal neurons treated with ethanol (0–300 mM) revealed a dose-dependent reduction in the levels of cell viability. 200 mM of ethanol caused about 30% hippocampal neurons apoptosis. Therefore, the addition of 200 mM ethanol was used in the subsequent experiments. Our results also revealed that carvacrol (0–0.8 mM) did not exhibit a significant toxic effect on the neurons (Figure 6(b)). As shown in Figure 6(c), 0.8 mM of carvacrol significantly increased cell viability compared with ethanol group (*P* < 0.01). Thus, the administration of 0.8 mM of carvacrol was used in the subsequent experiments. This result was confirmed by Hoechst 33342 staining (Figure 6(d)).

### 3.4. Carvacrol Inhibited Oxidative Stress in Hippocampus of Ethanol-Fed Mouse

**Figure 7:**
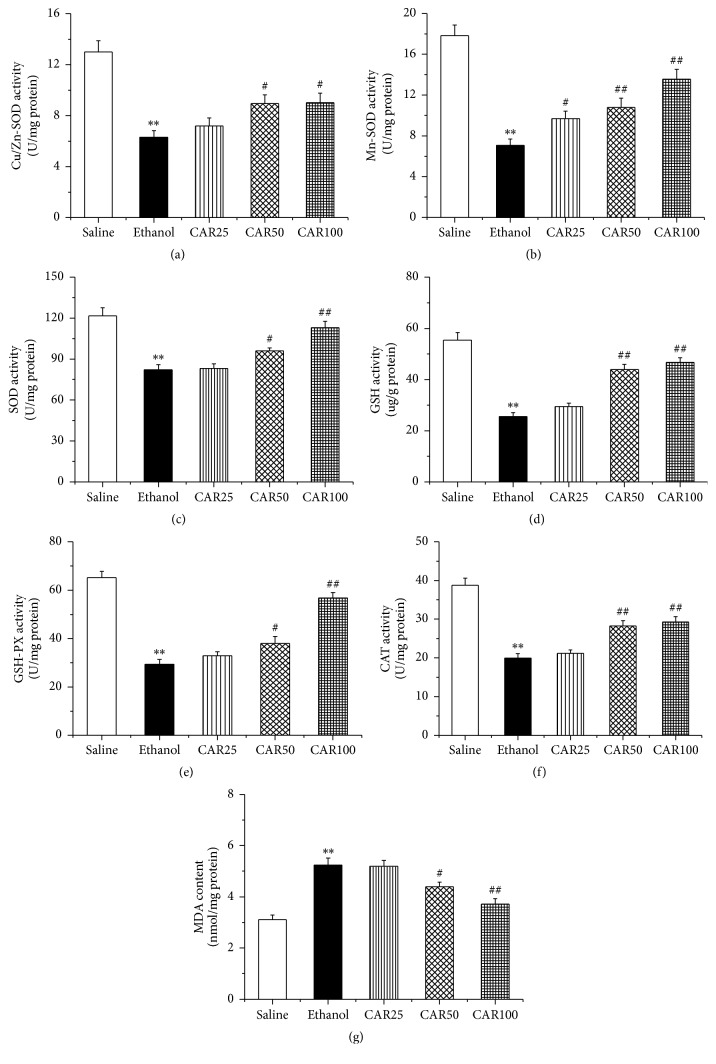
Effects of carvacrol on oxidative stress biomarkers of the hippocampus in ethanol-treated C57BL/6 mice. (a)–(g) showed the effects of carvacrol on activities of CuZn-SOD, Mn-SOD, SOD, GSH, GSH-PX, CAT, and MDA level from different group, respectively. Saline: saline group; Ethanol: ethanol group; CAR25: ethanol and carvacrol 25 mg/kg-treated group; CAR50: ethanol and carvacrol 50 mg/kg-treated group; CAR100: ethanol and carvacrol 100 mg/kg-treated group. The data are expressed as the mean ± SEM (*n* = 6 per group). ^*∗∗*^*P* < 0.01 compared to saline group; ^#^*P* < 0.05 and ^##^*P* < 0.01 compared to ethanol group.

To explore whether carvacrol protects the neuronal impairment via antioxidant mechanisms, oxidative stress biomarkers were evaluated in our study. As shown in Figure 7, our results indicated that the ethanol exposure reduced the activities of the antioxidative enzymes and antioxidants compared to the saline group (*P* < 0.01), including CuZn-SOD, Figure 7(a), Mn-SOD, Figure 7(b), SOD, Figure 7(c), GSH, Figure 7(d), GSH-PX, Figure 7(e), and CAT, Figure 7(f). Treatment with carvacrol at both of 50 and 100 mg/kg significantly reversed the above changes. The concentration of MDA, an index of lipid peroxidation, is depicted. As shown in Figure 7(g), the level of MDA in the ethanol group was significantly increased compared with that of the saline group (*P* < 0.01). Treatment with carvacrol at doses of 50 and 100 mg/kg suppressed the levels of MDA.

### 3.5. Carvacrol Increases Bcl-2 Protein Level in Hippocampal Neurons under Ethanol Stimulation

**Figure 8:**
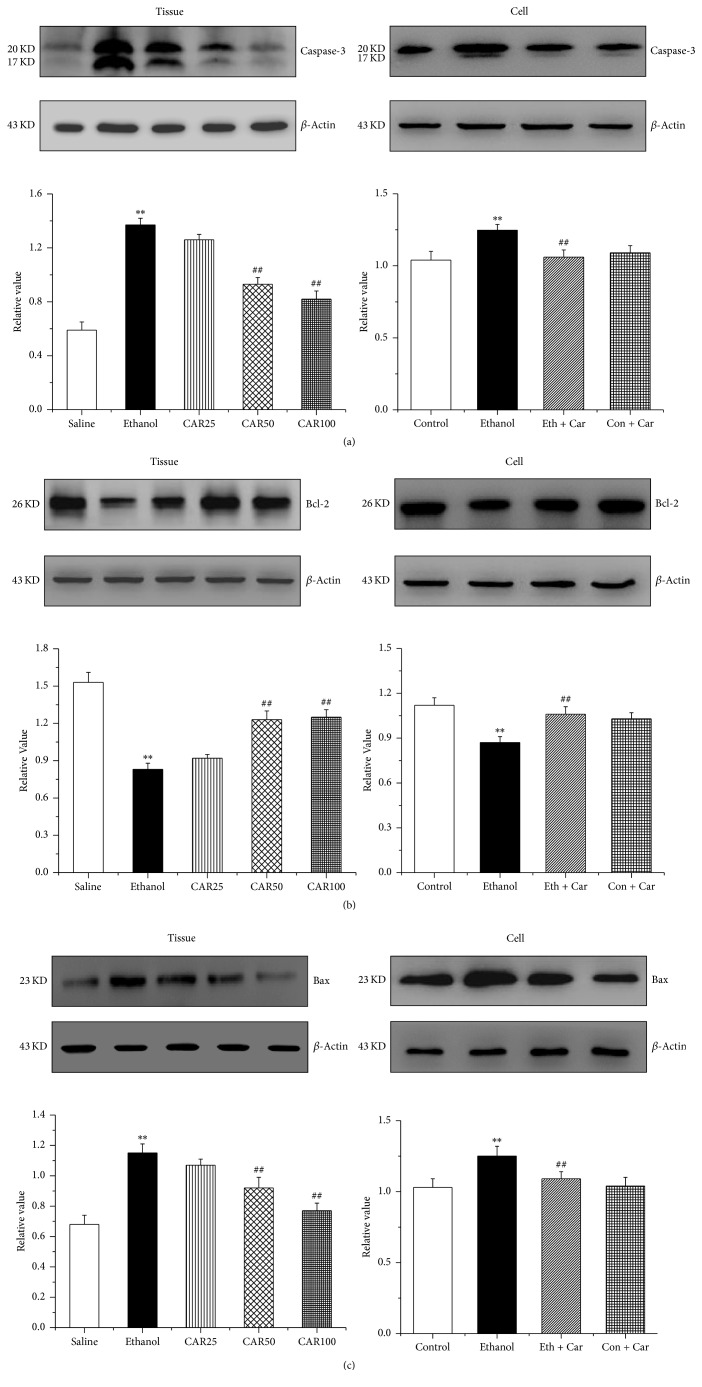
Carvacrol altered the protein expression of caspase-3, Bcl-2, and Bax of the hippocampus in ethanol-treated C57BL/6 mice and hippocampal neurons. (a), (b), and (c) show the quantitative analysis of the protein levels of caspase-3, Bcl-2, and Bax in mouse hippocampi and hippocampal neurons, respectively. The data were normalized to the loading control GAPDH. The data are expressed as the mean ± SEM (*n* = 6 per group). ^*∗∗*^*P* < 0.01 compared to saline group; ^##^*P* < 0.01 compared to ethanol group.

To investigate whether carvacrol modulated the mitochondrial pathway, the expressions of apoptosis-regulatory indices, including caspase-3, Bcl-2, and Bax were detected by western blot. Our results showed that the expressions of caspase-3 and Bax were remarkably increased but Bcl-2 was decreased in ethanol-treated mice compared with the saline group (*P* < 0.01). Administration of 50 and 100 mg/kg carvacrol decreased Bax and caspase-3 expression but increased Bcl-2 protein levels compared with the ethanol-treated mice (*P* < 0.01) (Figures 8(a), 8(b), and 8(c)).

### 3.6. Carvacrol Protects Hippocampal Neurons Apoptosis by MAPKs Signal Pathway

**Figure 9:**
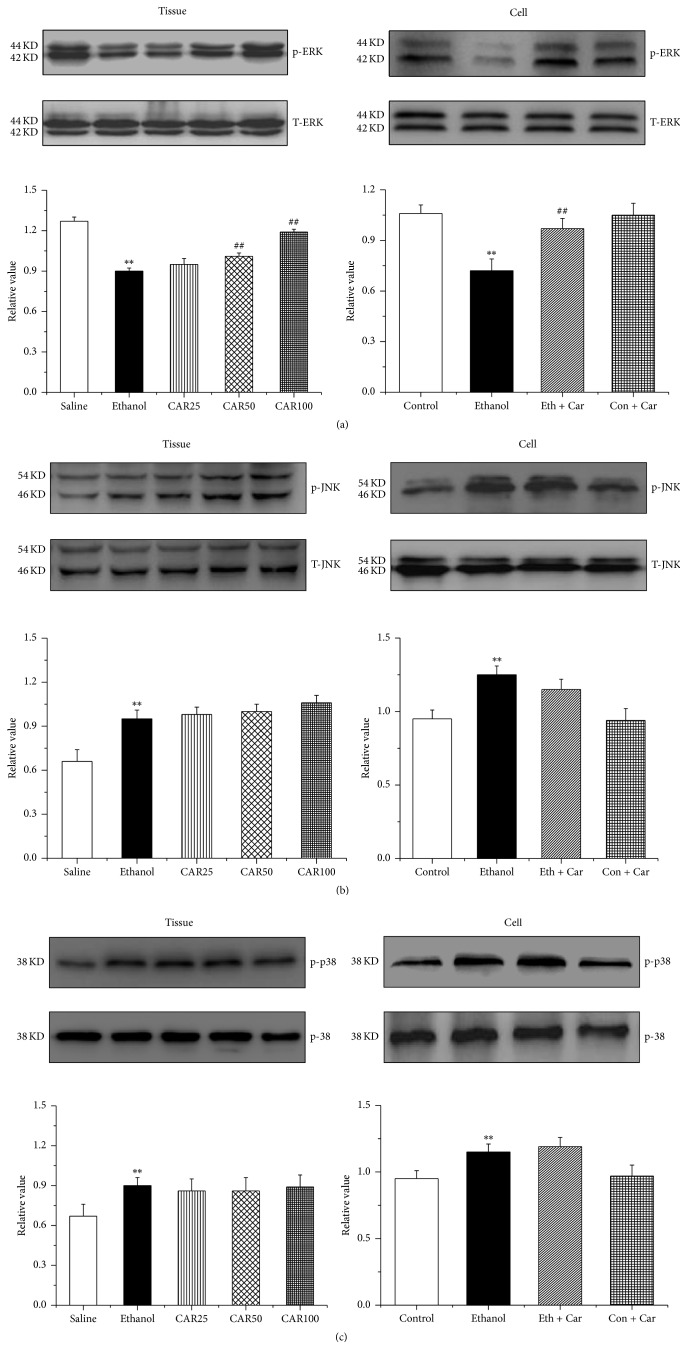
Carvacrol influenced the protein levels of MAPK cascades of the hippocampus of ethanol-treated C57BL/6 mice and ethanol-treated hippocampal neurons. (a), (b), and (c) are the quantitative analyses of the protein levels of p-ERK-1/2, p-JNK, and p-p38, which were normalized to ERK-1/2, JNK, and p38, respectively. The data are expressed as the mean ± SEM (*n* = 6 per group). ^*∗∗*^*P* < 0.01 compared to saline group; ^##^*P* < 0.01 compared to ethanol group.

To assess the possible involvement of MAPK activation in the effects of carvacrol on ethanol-induced impairment, we first examined MAPK signal activation in hippocampus by western blotting analysis. As shown in Figure 9(a), ethanol exposure reduced ERK-1/2 protein phosphorylation (p-ERK-1/2). p-ERK-1/2 was remarkably elevated in the mice treated with carvacrol at doses of 50 and 100 mg/kg in comparison to the ethanol group. As indicated, the phosphorylation of JNK, Figure 9(b), and p38, Figure 9(c), was markedly increased in ethanol-treated mice and hippocampal neurons, compared to the saline group. However, the carvacrol did not reverse the effect of ethanol on phosphorylation of JNK and p38.

**Figure 10:**
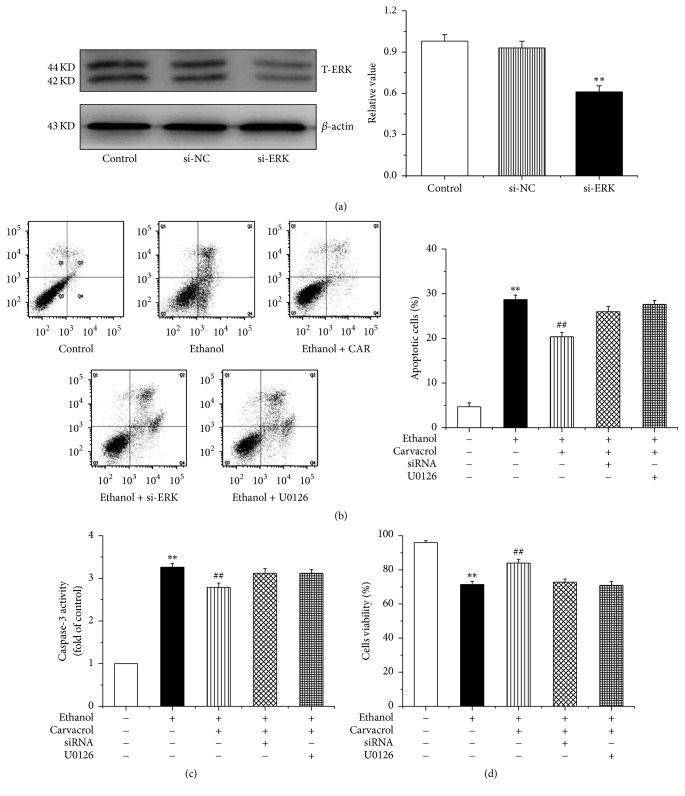
Silence ERK pathway partially repeal the antiapoptosis effects of carvacrol. (a) The efficiency and specificity of siRNA directed against ERK. (b)–(d) Ethanol increases hippocampal neurons apoptotic rate and caspase-3 activity as well as decreasing cell viability compared with control group. These proapoptotic effects can be reversed by carvacrol. However, when it is silence or block ERK pathway, carvacrol antiapoptotic effects can be partially repealed. The data are expressed as the mean ± SEM (*n* = 6 per group). ^*∗∗*^*P* < 0.01 compared to control group; ^##^*P* < 0.01 compared to ethanol group.

We further silenced ERK1/2 (Figure 10(a)) to confirm carvacrol protective effects through ERK1/2 signaling pathway. Our results revealed that carvacrol significantly decreases hippocampal neurons apoptosis compared to the ethanol group (*P* < 0.01), but these effects can be reversed by silence of ERK1/2 or treatment of U0126 (antagonist of carvacrol) (Figure 10(b)). The caspase-3 activity and cell viability change consistently with the result of hippocampal neurons apoptosis (Figures 10(c) and 10(d)). Taken together, these findings suggested that carvacrol protects hippocampal neurons apoptosis by ERK1/2 signal pathway.

## 4. Discussion and Conclusion

In this study, we demonstrated that carvacrol improves ethanol-mediated cognitive dysfunction of the mice and then alleviates hippocampal neuronal impairment by antioxidative and antiapoptotic effects, which is likely related to the mitochondrial and mitogen-activated protein kinase signal pathway.

The ethological [32], morphological [33], chemical, and molecular biological determinations showed that exposure to ethanol for 4 weeks can induce oxidative stress [34] and result in mice hippocampal neuronal apoptosis [35], which finally leads to cognitive dysfunction [36]. Our study showed that carvacrol treatment partially reversed learning and memory deficits in the ethanol-induced model by decreasing escape latency and increasing number of times of platform passing. At the same time, Nissl staining and NeuN immunohistochemistry showed that carvacrol treatment at doses of 50 and 100 mg/kg significantly reduced the neuronal apoptosis in vivo. Furthermore, 0.8 mM of carvacrol significantly improves cell viability of ethanol-induced hippocampal neuron in vitro. These findings indicate that carvacrol alleviates learning and memory deficits via inhibiting hippocampal neuronal apoptosis.

The compelling evidence supported that oxidative damage, an important index for evaluating the neuronal injury caused by ethanol, plays a crucial role in neuropathologic lesions [37–39]. Therefore, we speculate that carvacrol partially reversed neuronal injury in the ethanol-induced model by decreasing hippocampal neuronal oxidative stress. Our results illustrated that the CuZn-SOD, Mn-SOD, SOD, GSH, GSH-PX, and CAT activities decreased and the level of MDA increased in ethanol-treated mice, and carvacrol partially reversed the changes. Therefore, the reduction of oxidative stress and enhancement of antioxidative stress might have an important role in the protective effects of carvacrol on ethanol-induced neuron injury.

It is well known that oxidative stress can induce apoptosis [40]. However, apoptosis pathways include the mitochondrial pathway, the death receptor pathway, and the endoplasmic reticulum stress pathway. We next sought to examine the signaling pathways that participated in the induction of hippocampal neuronal apoptosis induced by ethanol. The mitochondrial pathway of apoptosis began with the permeabilisation of the mitochondrial outer membrane. Furthermore, mitochondrial membrane permeabilisation is regulated by Bcl-2 family members, which can be subdivided into antiapoptotic members such as Bcl-2 and proapoptotic species such as Bax [41]. In our study, high concentration of ethanol could upregulate the expression of Bax and downregulate the expression of Bcl-2. Carvacrol could further weaken the effect of ethanol. Thus, it was speculated that carvacrol protects hippocampal neuron from apoptosis through mitochondrial pathway associated with the upregulation of antiapoptotic protein Bcl-2 and downregulation of proapoptotic protein Bax.

Alteration of the MAPKs signal pathway has been observed in ethanol-induced cell death [42, 43]. We found that ethanol inhibited the phosphorylation of ERK-1/2, which is in agreement with previous research in which ethanol exposure decreased ERK phosphorylation [44] but increased that of p-JNK and p-p38 in ethanol-treated mouse hippocampus [22]. However, carvacrol reversed the phosphorylation of ERK-1/2 change induced by ethanol and had no influence on p-JNK and p-p38 expression. The results indicate that activation of ERK-1/2 signaling pathway is a possible neuroprotective mechanism of carvacrol following ethanol exposure.

Our data in the present study indicate that the protective effects of carvacrol on ethanol-induced cognitive dysfunction of the mice and hippocampal neuronal impairment might be associated with antioxidative and antiapoptotic effects as well as the modulation of MAPK cascades and mitochondrial pathway. Since carvacrol is a natural product with a proven record of safe human administration, it is possible that carvacrol may be regarded as a promising drug in clinical therapy.
